# Middle meningeal artery embolization in chronic subdural hematoma: bridging surgical innovations and molecular mechanisms

**DOI:** 10.3389/fsurg.2026.1756744

**Published:** 2026-07-02

**Authors:** Rasit Dinc, Nurittin Ardic

**Affiliations:** 1 INVAMED Medical Innovation Institute, New York, NY, United States; 2 Med-International UK Health Agency Ltd., Nuneaton, United Kingdom

**Keywords:** angiogenesis, artificial intelligence, biomarkers, chronic subdural hematoma, hyperfibrinolysis, inflammation, middle meningeal artery embolization, precision medicine

## Abstract

**Background:**

Chronic subdural hematoma (CSDH) is one of the most common neurosurgical conditions in the elderly. Despite advances in burr-hole craniotomy, up to one-third of patients experience clinically significant recurrences, and most are not suitable candidates for repeat surgery.

**Objective:**

To summarize current surgical concepts and clinical evidence for middle meningeal artery embolization (MMAE) in CSDH and integrate this data with our current understanding of the molecular and cellular mechanisms that drive disease persistence and response to treatment.

**Methods:**

We detail the epidemiology and standard surgical management of CSDH, discuss prospective and randomized evidence for the use of MMAE as adjunctive, primary, or salvage therapy, and synthesize experimental and clinical data on inflammation, angiogenesis, and coagulation in the CSDH neomembrane. We then summarize mechanistic hypotheses regarding how MMAE alters this microenvironment and highlight future directions, including biomarker development and AI-assisted precision therapy. Unlike previous reviews that focused primarily on clinical outcomes, this article highlights the biological mechanisms underlying MMAE efficacy and their impact on precise patient selection.

**Results:**

Recent randomized controlled trials of MMAE have yielded mixed but encouraging results. Some trials have shown a reduction in relapse or treatment failure with adjunctive MMAE, whereas others have failed to achieve primary endpoints. Heterogeneity in study design, patient selection, and outcome definitions limits direct comparison. Mechanistic studies demonstrate that CSDH is a vascularized and inflamed microenvironment nourished by fragile MMA-derived vessels. Embolization may reduce microbleeding, dampen inflammation, and promote hematoma resorption.

**Conclusion:**

By targeting underlying vascular and inflammatory processes, MMAE is a promising adjunct and, in selected cases, an alternative strategy for CSDH. However, current evidence is heterogeneous, and further studies are needed to define its safety, efficacy, and cost-effectiveness. Integration of mechanistic information with biomarker and imaging-based approaches may improve patient selection and treatment strategies.

## Graphical Abstract

**Figure:**
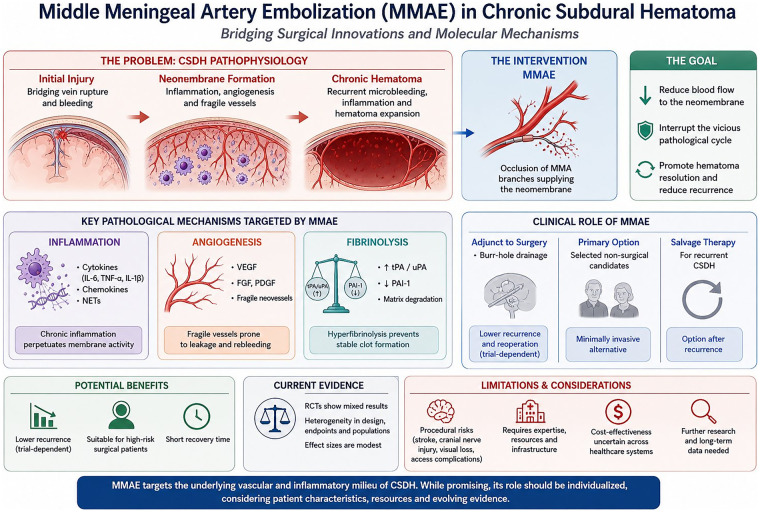
Conceptual overview of middle meningeal artery embolization (MMAE) in chronic subdural hematoma (CSDH). The left panel illustrates the pathophysiological progression of CSDH from initial bleeding to neomembrane formation and chronic hematoma expansion, driven by inflammation, angiogenesis, and dysregulated fibrinolysis. The central panel highlights the mechanism of MMAE, involving occlusion of the middle meningeal artery branches supplying the neomembrane and potentially reducing pathological vascular supply. The right and lower panels summarize the clinical role of MMAE as adjunctive to surgery, a primary option in selected patients, or salvage therapy, along with potential benefits, and current limitations. While some randomized trials suggest a reduction in recurrence or treatment failure, results remain heterogeneous, and effect sizes are modest. Implementation is influenced by procedural risks, resource requirements, and cost considerations. Overall, MMAE represents a promising but emerging strategy that could complement current therapies. The most appropriate role will likely depend on patient selection, clinical context, and further high-quality evidence.

## 1 Introduction

Chronic subdural hematoma (CSDH) is one of the most common neurosurgical pathologies in aging populations, and its incidence is projected to double by 2030 as demographics shift toward older age groups (1). This condition develops when blood accumulates in the subdural space following minor trauma, forming an encapsulated collection characterized by vascularized membranes, ongoing inflammation, and recurrent microhemorrhage (2). This chronic course distinguishes CSDH from acute hemorrhage and explains its progressive expansion over weeks or even months.

Burr-hole craniotomy with drainage remains the standard surgical treatment, but recurrence rates of 10%–30% pose a persistent clinical challenge (3). Recurrence often necessitates repeated interventions in elderly patients with multiple comorbidities and anticoagulation requirements. Middle meningeal artery embolization (MMAE) has emerged as a minimally invasive surgical alternative or adjunctive treatment targeting arterial blood supply to pathological neomembranes (4).

Recent randomized controlled trials (RCTs) have significantly improved the evidence base for MMAE, but the overall picture is more complex than a uniformly positive treatment effect. The EMBOLISE and STEM studies reported lower rates of composite treatment failure or reoperation-related outcomes with MMAE, whereas the MAGIC-MT and EMPROTECT studies did not show a statistically significant reduction in primary recurrence endpoints. Inter-study interpretation is complicated by heterogeneity in inclusion criteria, use of surgical and non-surgical standard care, embolic materials, follow-up intervals, and endpoint definitions. Accordingly, MMAE should currently be viewed as a promising but still evolving treatment strategy, and its precise role in routine care is yet to be defined (5–8). Despite growing clinical evidence, the molecular mechanisms by which MMAE promotes hematoma resolution are not yet fully understood.

The pathophysiology of CSDH involves complex molecular processes such as persistent inflammation, pathological angiogenesis, hyperfibrinolysis, and immune dysregulation (2). The subdural space becomes a metabolically active microenvironment where inflammatory cytokines, growth factors, and proteases form self-perpetuating cycles that impede normal healing. Understanding how MMAE may interact with these pathological cascades is important for optimizing patient selection, predicting treatment response, and developing complementary therapies.

Artificial intelligence (AI) approaches are increasingly being applied to neurosurgical outcome prediction by integrating clinical, imaging, and molecular data to guide personalized treatment decisions (9, 10). For CSDH, where patient heterogeneity and variable treatment responses are significant, AI-enabled precision medicine can improve outcomes by matching patients with the most appropriate interventions based on their individual risk profiles.

This review examines the molecular mechanisms underlying the pathophysiology of CSDH and provides a mechanistic framework for MMAE efficacy. We analyze the inflammatory, angiogenic, and coagulation processes that drive hematoma persistence, describe how MMAE may influence these pathways, and explore future directions, including biomarker development and AI-assisted precision therapy. By focusing on the interface between detailed pathophysiology and interventional technique, we aim to complement, rather than replicate, existing randomized evidence syntheses in this research topic.

### 1.1 Review methodology

This article is designed as a narrative review rather than a systematic review or meta-analysis. Relevant literature was identified through targeted searches in PubMed/MEDLINE and Google Scholar and supported by a reference search of significant reviews, randomized trials, and large observational studies. Search terms included “chronic subdural hematoma,” “middle meningeal artery embolization,” “MMA embolization,” “inflammation,” “angiogenesis,” “fibrinolysis,” “biomarkers,” “liquid biopsy,” and “artificial intelligence.” Priority was given to randomized controlled trials, systematic reviews, large observational cohorts, and mechanistic studies directly relevant to the pathophysiology of CSDH or MMAE. Because this is a narrative synthesis, formal risk-bias scoring and quantitative pooling were not performed. The aim is to integrate clinical evidence with translational mechanisms while explicitly acknowledging areas where the evidence remains preliminary or hypothesis-generating.

## 2 Surgical techniques and current evidence—a brief overview

Burr-hole craniotomy, which involves creating one or two burr holes through which the hematoma is drained by irrigation, remains the most widely used surgical technique for CSDH (11). A landmark study by Santarius et al. (11) demonstrated that placement of a subdural drain significantly reduced recurrence from 24% to 9%, making drainage standard practice. Despite surgical improvements, recurrence rates remain at 10%–20%, influenced by patient factors such as anticoagulation use, bilateral hematomas, and comorbidities (12).

MMAE offers a minimally invasive endovascular approach that targets the middle meningeal artery branches supplying the pathological neomembrane. The procedure involves femoral or radial artery access, microcatheter advancement into the MMA branches, and selective embolization using particles, liquid embolic agents, coils or combined approaches depending on the anatomy, the practitioner's preference, and whether the vascular occlusion is distal or proximal (13, 14). Technical success exceeds 95% in most series, and the procedure typically takes 30–60 min under conscious sedation. A recent narrative review by Dinc summarized early clinical experience and technical considerations for MMAE in CSDH, emphasizing its minimally invasive profile and potential to reduce recurrence in high-risk patients (13).

Clinical evidence has rapidly evolved from retrospective to randomized trials, but the randomized evidence remains mixed and should be interpreted cautiously. In the EMBOLISE study, adjuvant MMAE plus surgery reduced recurrence or progression leading to repeat intervention compared to surgery alone (5). In the STEM study, adjuvant MMAE reduced the combined treatment failure outcome without increasing the short-term disabling stroke or mortality rate (6). In contrast, MAGIC-MT did not show a statistically significant reduction in symptomatic recurrence or progression within 90 days compared to conventional care (7), and EMPROTECT did not statistically significantly reduce 6-month recurrence after surgery in high-risk patients (8). These studies differ significantly in terms of eligibility criteria, use of standard non-surgical care vs. surgery, embolic materials, and endpoint formation, making inter-study comparison difficult. In particular, differences in trial design, including the adjunctive vs. standalone use of MMAE, variations in embolic techniques, and heterogeneity in endpoint definitions, limit direct comparison and interpretation. A recent systematic review and meta-analysis by Wach et al. provides a useful synthesis of the emerging study literature, but the primary studies themselves remain heterogeneous (15). Therefore, the current RCT literature supports MMAE as a promising approach, particularly in selected patients, but not yet uniformly established standard care across CSDH scenarios. In current practice, MMAE can be used as an adjunct to surgery, as a standalone option in selected high-risk or non-surgical patients, and as salvage therapy for recurrent hematomas (16).

From a practical standpoint, MMAE also has important resource and implementation considerations that should be acknowledged. Compared to burr-hole drainage alone, MMAE requires endovascular expertise, angiography suite availability, specialized embolic materials, additional procedural coordination, and exposure to contrast agent and radiation. Direct procedural costs are generally higher than standard surgery, but some studies suggest that total hospital-stay and follow-up costs may become more comparable after adjusting for confounding factors (16). More recent economic analyses suggest that MMAE is unlikely to be universally cost-effective across all healthcare systems and may be most appropriate in select patients with a higher risk of recurrence or reoperation (17).

Technical success is graded from 0 (no embolization) to 3 (complete embolization of the anterior and posterior MMA branches), with Grade 3 being associated with optimal outcomes (18). Radiographic hematoma resolution, defined as a greater than 75% reduction in volume, occurs in 90% of cases within six months, with functional improvement (mRS 0-2) achieved in 76% of patients (19). Reported complication rates are generally low, but the risk profile should be carefully considered. Potential adverse events include ischemic stroke or transient ischemic attack due to reflux or off-target embolization, cranial nerve damage via dangerous anastomoses, vision loss due to ophthalmic reflux, scalp ischemia or necrosis, access site complications such as groin or radial hematoma and pseudoaneurysm, and contrast-induced reactions or nephropathy (13, 14). These risks reinforce the importance of careful angiographic evaluation, experienced operators, and judicious patient selection.

Despite growing clinical interest and encouraging results in some studies, the molecular mechanisms by which MMAE promotes hematoma resolution still require further elucidation to optimize patient selection and develop complementary therapies.

## 3 Molecular mechanisms driving CSDH pathophysiology

### 3.1 Inflammation and neomembrane formation

CSDH pathophysiology is driven by persistent inflammation that fails to transition to normal wound healing (2). Following the initial hemorrhage, inflammatory mediators attract immune cells to the subdural space, creating a chronic inflammatory microenvironment. Proinflammatory cytokines, including interleukin-6 (IL-6), interleukin-8 (IL-8), and tumor necrosis factor-alpha (TNF-α), are significantly elevated in CSDH fluid compared to serum, suggesting local production (2, 20).

IL-6 promotes inflammatory cell recruitment, increases vascular permeability, and stimulates angiogenesis. Studies show that IL-6 levels are associated with hematoma volume and risk of recurrence (2). IL-8 acts as a potent neutrophil chemotactic agent, while TNF-α activates endothelial cells and increases permeability. Despite the presence of anti-inflammatory cytokines such as IL-10, the balance remains tilted toward net inflammation, which impedes healing (21).

IL-1β, another important pro-inflammatory cytokine elevated in CSDH fluid, amplifies the early inflammatory response by activating endothelial cells and increasing leukocyte recruitment, thus promoting the transition from acute hemorrhage to chronic inflammation. Conversely, transforming growth factor-*β* (TGF-*β*) promotes fibroblast proliferation and extracellular matrix deposition, contributing to neomembrane fibrosis and structural maturation that perpetuate the chronic disease state (22). The inflammatory cascade drives neomembrane formation. These vascularized capsules develop within 1–3 weeks, consisting of a dura-bound outer membrane containing proliferating fibroblasts, inflammatory cells, and immature blood vessels (23). Macrophages within the membranes phagocytose blood breakdown products but secrete additional inflammatory mediators and growth factors, creating self-reinforcing loops (24). The phenotypic shift toward pro-inflammatory M1 macrophages rather than pro-resolution M2 macrophages impedes normal healing transitions. Similar observations in intracerebral hemorrhage suggest that therapeutic modulation of macrophage polarization may accelerate hematoma resolution and provide a conceptual template for similar strategies in CSDH (25). Recent studies also add another layer of immune dysregulation by highlighting the role of neutrophil extracellular traps (NETs) in perpetuating inflammation and promoting immature angiogenesis (22).

### 3.2 Angiogenesis and membrane vascularization

Pathological angiogenesis represents a critical feature of CSDH, with neomembranes containing numerous fragile, thin-walled vessels prone to spontaneous rupture, which is the source of ongoing microbleeding that leads to dilation (2). These vessels receive blood flow predominantly from MMA branches traversing the dura (26).

Vascular endothelial growth factor (VEGF), present at significantly elevated levels in CSDH fluid and membrane tissue, serves as the primary angiogenic driver (27). VEGF stimulates endothelial proliferation, migration, and tube formation but produces structurally abnormal vessels lacking the normal hierarchy, with incomplete connections, a disrupted basement membrane, and inadequate pericyte coverage (28). This immature structure makes the vessels mechanically fragile and hyperpermeable, allowing plasma protein extravasation that contributes to osmotic fluid accumulation.

Angiopoietin-Tie2 signaling regulates vascular maturation. CSDH membranes exhibit relatively low angiopoietin-1 and high angiopoietin-2 levels, creating an imbalance that promotes immature, unstable vessel formation (27, 29). Matrix metalloproteinases (MMPs), particularly MMP-2 and MMP-9, facilitate angiogenesis by degrading the extracellular matrix and increasing permeability. MMP levels are associated with hematoma volume and risk of recurrence (28, 30).

The disorganized neovascular network—torn, dilated, and chaotically interconnected—lacks efficient flow dynamics and mechanical integrity. Importantly, these vessels receive their blood supply predominantly from the MMA branches, providing the anatomical rationale for embolization therapy (31).

### 3.3 Coagulation-fibrinolysis dysregulation

CSDH exhibits a paradoxical coagulation-fibrinolysis imbalance that prevents normal clot formation while preserving a fluid hematoma amenable to expansion (23). Tissue plasminogen activator (tPA) and urokinase-type plasminogen activator are elevated in CSDH fluid and convert plasminogen to plasmin, which breaks down fibrin clots (32). This hyperfibrinolytic environment produces fibrin degradation products that exert osmotic effects and draw fluid into the subdural space. Plasminogen activator inhibitor-1 (PAI-1), which normally regulates fibrinolysis, appears to be relatively insufficient compared with plasminogen activators in CSDH, resulting in net hyperfibrinolytic activity (33). Minor bleeding episodes from fragile neomembrane vessels deposit fibrin, but hyperfibrinolysis dissolves clots before significant clot formation occurs, inhibiting the normal healing process.

Anticoagulant and antiplatelet medications significantly impact CSDH. Direct oral anticoagulants and antiplatelet agents increase both the initial risk of CSDH and the likelihood of recurrence (1). Management of these medications presents therapeutic dilemmas, as discontinuation carries a thromboembolic risk, while continuation promotes bleeding. This challenge has increased interest in MMAE as a strategy to ensure safer medication continuation.

## 4 Mechanical basis of MMAE effectiveness

### 4.1 Impairment of vascular supply

MMAE provides therapeutic benefit primarily by devascularizing the pathological neomembrane. The concept that MMA branches nourish the outer membrane was first proposed by Mandai et al. (26) in 2,000, and subsequent angiographic studies have detailed that MMA branches penetrate the dura mater and supply the highly vascularized outer membrane (31). By occluding these feeding arteries, MMAE reduces or interrupts blood flow to the delicate capillary networks responsible for ongoing microbleeding.

Post-embolization imaging studies reveal a progressive decrease in neomembrane retention and vascularity (19). Serial angiography demonstrates the disappearance of the hypervascular staining pattern characteristic of active CSDH membranes following successful embolization (34). Without arterial blood flow, immature vessels within the membrane regress due to lack of perfusion pressure and nutrient depletion, interrupting the hemorrhage-inflammation-angiogenesis cycle.

The degree of vascular obstruction is associated with clinical outcomes. Complete embolization (Grade 3) provides better hematoma resolution and lower recurrence compared to partial embolization (16, 35). This dose-response relationship supports vascular devascularization as the primary treatment mechanism.

An additional mechanistic uncertainty concerns how the degree and level of MMA occlusion affects effectiveness. Distal embolization with particles or liquid agents may better penetrate membrane-feeding branches, while proximal occlusion with coils may be less effective if distal collateralization persists, although proximal interruption may still reduce flow in selected anatomies. Similarly, surgically interrupting dural feeders during craniotomy may share some of the same mechanistic logic. Currently, comparative evidence is insufficient to conclude that one occlusion strategy is universally superior, and this remains an important area for future studies (10, 13).

### 4.2 Modulation of the inflammatory microenvironment

Ischemic neomembrane tissue undergoes apoptosis and resorption, reducing the source of inflammatory cytokine production (36). Macrophages polarization within the membrane may shift toward a more pro-resolution profile after reduction of vascular supply, although direct longitudinal evidence after MMAE remains limited (37).

Decreased vascular permeability after embolization may reduce plasma protein extravasation, reducing the osmotic gradient that drives fluid accumulation (38). Reduced VEGF signaling, potentially related to hypoxia and inflammation, may decrease endothelial permeability, allowing for the reestablishment of normal subdural space physiology. The inflammatory environment may shift from chronic activation toward a more resolution-oriented phase, enabling normal wound healing processes. Decreased vascular permeability after embolization is expected to reduce plasma protein extravasation and suppress inflammatory signaling in the subdural space. Inflammatory markers such as IL-6 and IL-8 are associated with worse neurological outcomes and greater hematoma burden in CSDH, suggesting that interventions that successfully control the disease, whether surgery or MMAE, may secondarily normalize these mediators (2, 20). However, direct longitudinal data specifically on cytokine changes after MMAE remain limited and represent an important area for future translational research.

### 4.3 Promotion of hematoma organization and resorption

MMAE may facilitate hematoma organization through multiple mechanisms. Interruption of ongoing bleeding may allow the existing clot to stabilize without the ongoing fibrinolysis-inducing hemorrhage (4). The subdural accumulation transitions from an active bleeding state to a more stable state that allows for gradual resorption through hyperfibrinolysis.

If the inflammatory environment shifts toward resolution, macrophage-mediated clearance of blood breakdown products is accelerated (37). Without the constant microhemorrhage that regenerates the hematoma, arachnoid granulations can absorb subdural fluid, and the dura-arachnoid interface can reappear. Serial imaging demonstrates progressive reduction in hematoma volume, with 90% of patients achieving greater than 75% reduction within six months (19).

The neomembrane may undergo involution after embolization. Histological studies of recurrent CSDH membranes after MMAE show decreased cellularity, decreased vascularity, and increased fibrosis compared to pre-embolization membranes (39). This likely represents a transition from pathological granulation tissue toward more mature scar tissue, potentially reducing the source of bleeding.

### 4.4 Molecular predictors of treatment response

Emerging evidence suggests that molecular biomarkers may ultimately be helpful in predicting response to MMAE, but this has not yet been prospectively confirmed. Patients with higher baseline VEGF levels show greater hematoma volume reduction after embolization, potentially reflecting angiogenesis-mediated pathologies more susceptible to vascular intervention (28). Conversely, elevated fibrinolytic markers may identify patients requiring additional pharmacological treatment beyond embolization alone.

Genetic polymorphisms involving fibrinolytic pathways, including PAI-1 variants, have been implicated in hemorrhagic and thrombo-inflammatory cerebrovascular conditions and, although direct evidence in CSDH is limited, they may be biologically plausible candidates for future CSDH risk stratification (40).

**Figure 1:**
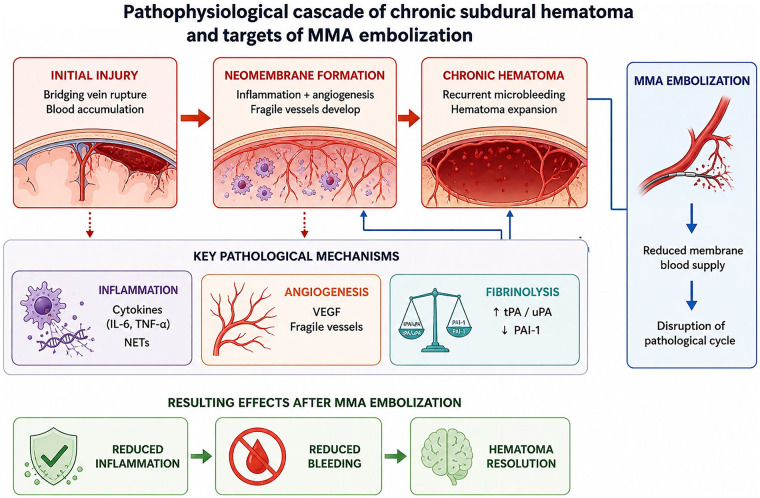
Pathophysiological cascade of chronic subdural hematoma and potential targets of middle meningeal artery embolization. CSDH typically develops following an initial bleeding event and leads to the formation of a new membrane characterized by inflammation, angiogenesis, and fragile neovascularization. This results in a self-sustaining cycle of microbleeding, fibrinolysis, and hematoma expansion. Key pathological processes include inflammatory activation (e.g., cytokine release and NETs), angiogenesis driven by VEGF, and dysregulated fibrinolysis with increased tPA/uPA and relatively decreased PAI-1. MMAE may reduce blood flow to the new membrane, thereby disrupting this pathological cycle. Downstream effects may include reduced inflammation, decreased recurrent bleeding, and facilitated hematoma resolution. These mechanisms are biologically plausible but have not yet been fully validated in prospective clinical trials. CSDH, chronic subdural hematoma; MMA, middle meningeal artery; MMAE, middle meningeal artery embolization; VEGF, vascular endothelial growth factor; Ang, angiopoietin; MMP, matrix metalloproteinase; tPA, tissue plasminogen activator; uPA, urokinase-type plasminogen activator; PAI-1, plasminogen activator inhibitor-1; FDPs, fibrin degradation products; NETs, neutrophil extracellular traps; TGF-*β*, transforming growth factor-beta.

Radiographic features predict MMAE efficacy. Hypervascular membrane involvement on angiography is associated with technical success and hematoma resolution (35). In contrast, organized, hypodense hematomas with minimal membrane vascularity show less benefit from MMAE, suggesting they represent advanced pathologies for which devascularization offers limited advantage (36). To visually integrate these interconnected processes, Figure 1 illustrates the pathological cascade of CSDH, and the key mechanistic control points targeted by MMAE.

## 5 Future directions and therapeutic implications

### 5.1 Optimizing MMAE based on molecular insights

A growing mechanistic understanding may help guide future treatment optimization, although many proposed applications remain investigational. Timing strategies can be tailored to the inflammatory stage; early MMAE to prevent membrane maturation during acute inflammation or delayed intervention for established hematomas (4). Molecular imaging using VEGF-targeted tracers may eventually help identify embolization candidates with highly angiogenic membranes most amenable to devascularization.

Combination approaches integrating MMAE with targeted molecular therapies are a promising area of development. Anti-inflammatory agents such as dexamethasone have shown preliminary efficacy in small CSDH but require validation (41). Tranexamic acid, an antifibrinolytic drug, has been shown to reduce hematoma growth in pilot studies and may complement MMAE by addressing hyperfibrinolysis (42). Combining vascular devascularization with pharmacological inflammation modulation may offer synergistic benefits, although this remains to be demonstrated in prospective studies.

Patient stratification based on molecular risk profiles may ultimately help optimize MMAE delivery, but this approach is still in the research phase. High-risk patients with elevated inflammatory markers, extensive membrane vascularity, or other features associated with relapse may prove to be suitable candidates for earlier MMAE use, but this biomarker-guided strategy has not yet been prospectively validated (35). Conversely, low-risk patients with minimal membrane involvement may achieve adequate outcomes with surgery, reserving MMAE for recurrence.

To avoid overstating the current role of MMAE, other recurrence prevention strategies should also be acknowledged. Antifibrinolytic therapy with tranexamic acid has shown promising but still developing evidence in CSDH, and local fibrinolytic-facilitated drainage strategies such as urokinase instillation have also been associated with lower recurrence in selected postoperative cases (43, 44). These approaches are mechanistically different from MMAE and may ultimately be complementary rather than mutually exclusive.

### 5.2 New therapeutic targets

Anti-angiogenic strategies targeting the VEGF pathway are conceptually appealing given the central role of pathological angiogenesis in CSDH, but clinical data in this context are currently lacking. Any future trials of VEGF-directed therapy will require a careful balance between potential benefits and risks such as impaired wound healing and thromboembolic events. Targeting matrix metalloproteinases may reduce membrane formation and vascular permeability. MMP inhibitors have shown promise in preclinical models, but careful clinical development is required given the essential roles of MMPs in normal physiology (30). Selective inhibition of MMP-2 and MMP-9, the isoforms most abundant in CSDH, may minimize off-target effects. Given the growing role of NETs in linking inflammation, coagulation, and vascular injury, pharmacological strategies that regulate NET formation or promote NET clearance (such as PAD4 inhibitors, DNase-based approaches, or agents that indirectly suppress NETosis) may be helpful in the future in treating MMAE in NET-high CSDH phenotypes. Our recent review of NETs in venous thromboembolism highlights how targeted suppression of excessive NET formation may mitigate thromboinflammatory damage in other vascular beds; this concept can also be applied to CSDH (22).

Regulating macrophage phenotypes toward pro-resolution M2 polarization may accelerate hematoma clearance. Agents that promote M2 polarization, such as IL-4 mimetics or peroxisome proliferator-activating receptor agonists, warrant investigation (45). One attractive concept is to use MMAE to shut down the vascular engine of neomembrane and then actively “reprogram” the remaining macrophage populations toward an M2-dominant, resolution-prone phenotype. Experimental and clinical data in intracerebral hemorrhage suggest that altering microglia/macrophage polarization can improve hematoma clearance and functional recovery (25), and similar immunomodulatory strategies, potentially administered systemically or locally via embolic carriers, are worthy of investigation in CSDH. A recent translational study in intracerebral hemorrhage supports the feasibility of macrophage-directed immunotherapy in hemorrhagic brain disease (22). Combining macrophage modulation with MMAE-induced devascularization is an intriguingly hypothesis, but it requires direct testing in CSDH models and clinical studies.

Statins exhibit pleiotropic anti-inflammatory and endothelium-stabilizing effects beyond lipid-lowering effects. Observational studies suggest that statin use is associated with reduced CSDH recurrence, potentially through membrane stabilization and decreased permeability (46, 47). Prospective studies evaluating statin add-on therapy are needed.

### 5.3 Biomarker development

The concept of liquid biopsy in CSDH necessitates a distinction between systemic blood sampling and localized hematoma fluid analysis. Localized samples taken during evacuation are likely to reflect neomembrane biology more directly because cytokines such as IL-6, IL-8, and VEGF are often more concentrated in hematoma fluid than in peripheral blood (20, 21). However, localized sampling is invasive, limited to surgically treated patients, and unlikely to be justified in all cases unless it alters treatment management. Peripheral blood biomarkers are less invasive and more scalable, but may be less specific and more susceptible to influence by factors such as age, concomitant diseases, systemic inflammation, and concomitant drug use. In the short term, biomarker-enriched sampling may be most rational in selected subgroups such as patients with recurrent disease, unusual radiographic behavior, inconsistent clinical-radiological evolution, or those participating in translational studies.

Molecular biomarkers may eventually contribute to CSDH management through improved prediction, monitoring, and treatment selection. Serum or CSDH fluid cytokine profiles (IL-6, IL-8, VEGF) may help stratify recurrence risk in future studies (2). High inflammatory markers may identify candidate subgroups for closer monitoring or future combination-treatment studies, while low markers indicate a favorable natural history with conservative treatment.

MMP levels are associated with hematoma volume and recurrence, suggesting their usefulness as prognostic biomarkers (30). Serial MMP measurement could potentially help monitor treatment response, but this requires validation. Fibrinolysis markers, including D-dimer and fibrin degradation products, can identify hyperfibrinolytic phenotypes requiring antifibrinolytic adjuvants.

Circulating endothelial cells and angiogenic progenitors released from neomembranes can be used as liquid biopsies reflecting membrane activity (48, 49). Quantification of these cells could enable noninvasive monitoring of membrane regression after MMAE. Cell-free DNA and microRNAs represent additional blood-based biomarkers that deserve investigation. Data from intracerebral and subdural hemorrhage models suggest that dysregulated microRNAs (e.g., elevated miR-21 and miR-146a, associated with neuroinflammation (50), and increased miR-144-5p, which promotes angiogenesis and vascular permeability in hematoma-derived exosomes (51, 52)) are promising biomarker and therapeutic candidates; however, systematic clinical validation in CSDH has not yet been performed. Advanced imaging biomarkers complement molecular markers. Quantitative MRI techniques, including diffusion-weighted imaging and perfusion imaging, characterize hematoma organization and membrane vascularity (53). Radiological features extracted from routine CT using machine learning can predict recurrence risk and MMAE response without additional imaging costs.

Currently, routine genotyping to predict MMAE response is not ready for clinical application. While candidate polymorphisms such as PAI-1 4G/5G are biologically interesting (40), there is insufficient evidence to support routine testing alone for treatment selection in CSDH. Given the costs, infrastructure requirements, and uncertain incremental predictive value compared to standard clinical and imaging variables, genotyping should be considered more of a research tool at this stage.

Biomarker and imaging signatures are also likely to be time-dependent. The biological profile of the hematoma probably evolves from early inflammatory activation to membrane maturation, angiogenesis, recurrent microhemorrhages, and in some cases, progressive organization. Accordingly, measurements obtained in the later stages of the disease course may not fully capture the previously dominant mechanisms. Whether such analytical methods can be applied early enough to predict progression toward chronicity in acute or early subacute subdural hemorrhage remains an open translational question and should be tested prospectively rather than hypothetically (2, 54).

Another unresolved issue is whether traumatic and seemingly spontaneous CSDH share the same biology. Both likely converge in common underlying mechanisms such as membrane formation, inflammation, angiogenesis, and fibrinolytic imbalance; however, the relative contribution of these pathways may differ depending on the initiating injury, the degree of brain atrophy, anticoagulant exposure, vascular fragility, and repeated microtrauma. Currently, the literature does not support treating traumatic and spontaneous CSDH as definitively distinct molecular entities, but this remains an important hypothesis for future studies (2, 53).

To summarize the key biological pathways involved in CSDH and their therapeutic implications, Table 1 provides an integrated overview of the major molecular mechanisms, biomarker candidates, and potential targets related to MMAE.

**Table 1:** Key molecular pathways in chronic subdural hematoma (CSDH): pathophysiological roles, biomarker potential, therapeutic implications, and representative references.

Pathway	Key molecules/cells	Pathophysiological role	Biomarker potential	Therapeutic implications	Representative references
Inflammation	IL-1β, IL-6, IL-8, TNF-α, IL-10	Sustained inflammatory signaling; endothelial activation; increased vascular permeability; recruitment of neutrophils and macrophages; failure to transition to the resolution phase	Elevated IL-6 and IL-8 are associated with hematoma volume, outcome, and risk of recurrence.	Anti-inflammatory agents (e.g., dexamethasone—under investigation; potential for targeted cytokine modulation	2,20,21
Neutrophil activation/NETs	Neutrophil extracellular traps (NETs)	Promotes persistent inflammation; stimulates immature angiogenesis; contributes to membrane instability.	Emerging biomarkers: NET-associated proteins (e.g., MPO-DNA complexes)	Potential future target for reducing inflammation and pathological angiogenesis	2,4,54
Angiogenesis	VEGF, Angiopoietin-2, Ang-1, MMP-2, MMP-9	Immature, leaky neovascularization; plasma extravasation; neomembrane hypervascularity	VEGF levels correlate with disease activity; MMP-2/9 predict recurrence	MMAE directly targets neovascular structures fed by MMA; anti-VEGF and MMP inhibitors may be used as adjunctive therapy in the future.	27–29
Fibrosis/membrane maturation	TGF-β, fibroblasts	Drives extracellular matrix accumulation; thickening and stabilization of neomembrane; maintains chronicity	Potential fibrotic markers; limited current clinical use	Modulation of fibroblast/TGF-β pathways may accelerate resolution after MMAE	21,27,29,39
Coagulation-fibrinolysis imbalance	tPA, uPA, plasmin, PAI-1, D-dimer, fibrin degradation products (FDPs)	Hyperfibrinolysis prevents clot stabilization; recurrent microhemorrhage accelerates hematoma expansion	D-dimer and FDPs reflect the hyperfibrinolytic phenotype; low PAI-1 increases the risk of recurrence	Antifibrinolytics (e.g., tranexamic acid, investigational); adjunctive role with MMAE	23,32,33
Macrophage polarization	M1 and M2 macrophage subsets	M1: proinflammatory; M2: reparative; imbalance contributes to chronic inflammation and delayed resolution	Macrophage-derived cytokine panels	Therapeutic strategies favoring the M2 phenotype may increase hematoma resolution	45,24
Genetic factors	PAI-1 4G/5G polymorphism	Affects postoperative fibrinolytic activity and risk of recurrence	Predictive polymorphism in surgical cohorts; significance for MMAE remains unknown	Genetic risk stratification may improve patient selection in the future	40
microRNAs	miR-21, miR-146a, miR-221	Regulates inflammation, angiogenesis, and fibroblast activity; reflects membrane activation status	Promising novel biomarkers for recurrence and treatment response; requires validation	Future integration into personalized treatment algorithms	50–52
Endothelial/progenitor markers	Circulating endothelial cells, angiogenic progenitors	Reflects membrane vascular activity and angiogenic drive.	Can be used as a liquid biopsy to monitor response to MMAE.	Potential for treatment monitoring and recurrence prediction	28,54
Imaging biomarkers	CT: membrane involvement, density patterns; MRI: perfusion, diffusion	Reflects membrane vascularity and hematoma organization; helps predict recurrence	Radiological and AI-derived imaging signatures demonstrate strong predictive value	Optimizes patient selection for surgery versus MMAE	10,35,36,53,55,56

Overall, biomarker-based stratification remains investigational. Prospective validation, standardized sampling protocols, and demonstration of incremental value beyond clinical and radiological predictors are required before routine clinical use.

### 5.4 Applications of artificial intelligence and precision medicine

The following AI and precision medicine concepts should be interpreted as forward-looking perspectives rather than established clinical tools for CSDH. AI may eventually enable the integration of clinical, imaging, and molecular data into predictive models, often outperforming traditional statistical approaches. Machine learning algorithms analyzing hospital admission CT scans predict recurrence with 80-85% accuracy, superior to clinical scoring systems (10, 56). Incorporating molecular biomarkers into these models may improve performance in future studies, but this has not yet been validated for routine clinical use.

Deep learning (DL) applied to CT and MRI can automatically segment hematomas, measure volumes, and characterize membrane properties, reducing interobserver variability and enabling objective longitudinal assessment (55). Automated analysis accelerates clinical workflows and provides quantitative endpoints for studies.

Natural language processing applied to electronic health records enables the generation of large-scale real-world evidence by extracting CSDH cases, treatment details, and outcomes from unstructured text (21). This data complements prospective registries and randomized trials, particularly for rare scenarios underrepresented in formal studies.

Decision support systems that integrate AI predictions with clinical judgment may eventually support treatment selection. Conceptually similar AI-driven decision frameworks have been proposed for intravascular device selection in aortic disease, demonstrating how multimodal clinical, imaging, and procedural data can be transformed into personalized treatment recommendations (57). Adapting such architectures to CSDH could enable dynamic selection between burr-hole surgery, MMAE, or combined strategies based on patient-specific risk profiles. For individual patients, algorithms could facilitate shared decision-making by predicting the likelihood of recurrence compared to surgery alone and MMAE combined (58). Such tools must undergo rigorous external validation and implementation science evaluation before being released for clinical use.

Precision medicine frameworks that classify CSDH into molecular subtypes, similar to cancer classifications, could refine future treatment strategies. Inflammatory-predominant, angiogenesis-predominant, and fibrinolysis-predominant subtypes may respond differently to targeted interventions and enable biomarker-guided therapy (2). Achieving this vision requires prospective multi-omics studies that integrate genomics, transcriptomics, proteomics, and metabolomics with clinical outcomes.

Building on AI frameworks initially developed for intravascular device selection in aortic disease (57), a similar multimodal decision engine for CSDH could be designed. Such a system could integrate clinical frailty scores, hematoma morphology, membrane vascularity, and molecular biomarkers (e.g., VEGF, MMPs, D-dimer) into personalized risk-benefit estimates for surgery alone, upfront MMAE, or combined strategies. Prospective evaluation of such AI-assisted treatment algorithms represents an important future direction for truly precision CSDH care.

### 5.5 Limitations of existing evidence

Several limitations should be considered when interpreting the current literature. First, existing randomized trials differ in terms of patient selection, comparator treatment, embolic agents, follow-up duration, and endpoint definitions, which limits direct inter-study comparison. Second, while many mechanistic explanations for MMAE, including inflammatory modulation, macrophage polarization, and biomarker-guided response prediction, are biologically plausible, they have not been fully validated in prospective clinical trials. Third, cost-effectiveness and resource utilization vary significantly among health systems, potentially limiting universal application. Finally, AI-based and biomarker-driven approaches are in the investigational phase and require external validation before being implemented in routine clinical practice.

## 6 Conclusion

Chronic subdural hematoma is a complex pathophysiological process driven by persistent inflammation, pathological angiogenesis, and dysregulated coagulation-fibrinolysis. Middle meningeal artery embolization is biologically attractive because it targets the vascular supply of the pathological neomembrane and may interrupt several mechanisms that sustain hematoma persistence. However, current randomized evidence is heterogeneous and should be interpreted with caution, particularly given differences in patient selection, study design, and outcome definitions. Currently, MMAE should be considered a promising adjunctive or selective alternative strategy rather than a universally established standard for all patients with CSDH. Future progress will depend on improved phenotyping, more robust translational biomarker studies, careful assessment of cost-effectiveness, and prospective validation of precision-treatment frameworks integrating clinical, imaging, and molecular data.
